# Pharmacokinetics and pharmacodynamics of intravenous delafloxacin in healthy subjects: model-based dose optimization

**DOI:** 10.1128/aac.00428-24

**Published:** 2024-06-20

**Authors:** Jiong-Xian Lv, Yi-Huan Huang, Farah Kafauit, Yuan-Hui Wang, Chang Su, Jun-Heng Ma, Yan Xu, Chao-Chao Huang, Qing Zhang, Yu-Wen Su

**Affiliations:** 1 School of Pharmacy, Nanjing Medical University, Nanjing, Jiangsu, China; 2 Department of Clinical Pharmacology, Sir Run Run Hospital, Nanjing Medical University, Nanjing, Jiangsu, China; Providence Portland Medical Center, Portland, Oregon, USA

**Keywords:** delafloxacin, bacterial skin infections, pharmacokinetics/pharmacodynamics, population pharmacokinetics, dose optimization

## Abstract

Delafloxacin, a fluoroquinolone antibiotic to treat skin infections, exhibits a broad-spectrum antimicrobial activity. The first randomized, open-label phase I clinical trial was conducted to assess the safety and pharmacokinetics (PK) of intravenous delafloxacin in the Chinese population. A population pharmacokinetic (PopPK) model based on the clinical trial was conducted by NONMEM software. Monte Carlo simulation was performed to evaluate the antibacterial effects of delafloxacin at different doses in different Chinese populations. The PK characteristics of delafloxacin were best described by a three-compartment model with mixed linear and nonlinear clearance. Body weight was included as a covariate in the model. We simulated the AUC_0-24h_ in a steady state at five doses in patient groups of various weights. The results indicated that for patients weighing 70 kg and treated with methicillin-resistant *Staphylococcus aureus* (MRSA) infections, a minimum dose of 300 mg achieved a PTA > 90% at MIC_90_ of 0.25 µg/mL, suggesting an ideal bactericidal effect. For patients weighing less than 60 kg, a dose of 200 mg achieved a PTA > 90% at MIC_90_ of 0.25 µg/mL, also suggesting an ideal bactericidal effect. Additionally, this trial demonstrated the high safety of delafloxacin in single-dose and multiple-dose groups of Chinese. Delafloxacin (300 mg, q12h, iv) was recommended for achieving optimal efficacy in Chinese bacterial skin infections patients. To ensure optimal efficacy, an individualized dose of 200 mg (q12h, iv) could be advised for patients weighing less than 60 kg, and 300 mg (q12h, iv) for those weighing more than 60 kg.

## INTRODUCTION

Acute bacterial skin and skin structure infections (ABSSSIs) represent a prevalent category of infections caused by bacteria. In the United States, the diagnostic rate of ABSSSIs has increased by nearly 3-fold among patients who visit emergency departments mainly due to skin conditions, such as abscesses and cellulitis. *Staphylococcus aureus*, particularly MRSA, assumes a pivotal role in the pathogenesis of ABSSSIs (1, 2). In China, inappropriate use of antibiotics, including excessive dosage and incorrect selection of antibiotics, has endowed a wide spectrum of bacteria with resistance (3). In concert with the growing prevalence of ABSSSIs and the abuse of antimicrobial agents, there is a pressing demand for novel therapeutic approaches (4–6).

Delafloxacin is a novel broad-spectrum fluoroquinolone antibiotic, developed by Japanese pharmaceutical company Wakunaga in collaboration with American Melinta Biosciences (7). It was approved for treating ABSSSIs by the Food and Drug Administration (FDA) in 2017 but has not been approved by National Medical Products Administration (NMPA) yet. Delafloxacin demonstrates a broad-spectrum antimicrobial activity against Gram-positive and Gram-negative bacteria, particularly the former, especially MRSA (8). The pharmacokinetics of intravenous delafloxacin in humans have been reported in two phase I clinical trials (5, 9), showing that delafloxacin-treated patients demonstrated a good tolerance and a low incidence of adverse reactions. The study results reveal uncertainty regarding the linear relationship of delafloxacin. The literature suggests that the lack of a clear linear relationship may be attributed to the presence of saturable elimination. In phase III clinical trials, delafloxacin at a recommended dosage of 300 mg q12h is considered the most effective, with efficacy comparable with mainstream market antimicrobial agents, like vancomycin and linezolid (10, 11). According to a meta-analysis of randomized controlled trials, the efficacy of delafloxacin is comparable with some other anti-MRSA antibiotics such as vancomycin, linezolid, and ceftobiprole for treating ABSSSIs (12, 13).

Two population pharmacokinetic (PopPK) models for delafloxacin have been established, both utilizing a three-compartment model with mixed linear and nonlinear clearance (14, 15). The fact that the model fitted very well indicated similarities in the pharmacokinetic characteristics of delafoxacin between healthy volunteers and patient subjects. According to the literature, delafloxacin has a free fraction of 0.16 (14–17). The free steady-state blood concentration-time curve area under the curve to minimum inhibitory concentration (*f*AUC/MIC) is used as the pharmacokinetics and pharmacodynamics (PK/PD) indicator associated with delafloxacin efficacy. Delafloxacin has shown a Minimum Inhibitory Concentration (MIC) value of approximately 1/2 to 1/4 of that of moxifloxacin in fighting against most Gram-positive bacteria (16, 17).

There was a lack of clinical trials related to delafloxacin and PK/PD characteristics in the Chinese population. Thus, we conducted a phase I clinical trial (CTR20213308) to investigate the PK/PD of delafloxacin in a Chinese population. Also, we built up a PopPK model using data from the clinical trial to better describe the PK/PD characteristics of delafloxacin in Chinese and its efficacy across various dosage regimens. This study offered recommendations for dosing delafloxacin across different patient groups.

## MATERIALS AND METHODS

### Study design

This open-labeled randomized trial was the first to expose the healthy Chinese population to delafloxacin. Single ascending doses at 150 mg, 300 mg, and 600 mg as well as multiple ascending doses at 300 mg were set in the trial. Additionally, a randomized, open-labeled, 2-period, cross-over trial was conducted to assess the bioequivalence between 300 mg delafloxacin and Baxdela (used as reference). The study utilized intravenous administration with a 1-h infusion duration. The dosing scheme is presented in the supplemental material.

### Subjects

According to the Guiding Principles in China for Clinical Pharmacokinetic Studies of Chemical Drugs and PK/PD Studies of Antimicrobial Drugs, 8–12 subjects were required for each dose group. A total of 60 subjects were enrolled in this trial. Single ascending dose study included 12 subjects, each dosed at both 150 mg and 600 mg, whereas the 300 mg single-dose group comprised 24 subjects. There were 12 subjects in the multiple ascending dose study. Subjects were able to effectively communicate with investigators, comprehend and adhere to the requirements of the study, understand the risks of the trial, and sign the informed consent form. Male and female patients aged 18 to 75 years, with men weighing ≥50 kg and women weighing ≥45 kg. The investigators assessed the overall good health of the subjects based on their medical history, physical examination, vital signs, 12-lead electrocardiogram, and laboratory test results. The exclusion criteria are presented in the supplemental material. All subjects provided informed consent prior to any study-related activity.

### Blood sample collection

In the single ascending dose study, the blood concentrations of delafloxacin were collected before and at 0.25, 0.5, 1, 1.25, 1.5, 2, 3, 4, 6, 8, 12, 16, 24, 36, and 48 h after dosing. In the multiple ascending dose study, blood was sampled before and at 0.25, 0.5, 1, 1.25, 1.5, 2, 3, 4, 6, 8, 12, 16, 24, 36, and 48 h after dosing; moreover, on the 4^th^ and 5^th^ day, blood samples were collected before dosing.

### Clinical data collection

We collected factors that might influence the PK profiles of delafloxacin. Demographic factors involved sex, age, height, body mass index (BMI), vital signs, physical and electrocardiogram examination; laboratory data covered white blood cells (WBC), red blood cells (RBC), albumin (ALB), alanine aminotransferase (ALT), aspartate transaminase (AST), serum creatinine (CREA), low-density lipoprotein (LDL), high-density lipoprotein (HDL), lactic dehydrogenase (LDH), total protein (TP), hemoglobin, platelets, neutrophil ratio, prothrombin time, prothrombin activity, and urine pH. Estimated glomerular filtration rate (eGFR) was calculated with a modified modification of diet in renal disease (MDRD) formula for Chinese and renal creatinine CL was calculated according to the Cockcroft–Gault (CG) formula.

### Bioanalytical method

All samples were centrifuged within 1 h after collection and stored at −20°C. The concentration of delafloxacin was quantified using liquid chromatography-tandem mass spectrometry (LC-MS/MS). The mobile phase A for delafloxacin detection was 100% water containing 10 mM ammonium acetate and 0.2% formic acid, whereas mobile phase B was 100% acetonitrile containing 0.2% formic acid. The internal standards for mass spectrometry were delafloxacin-d5(Lot No.4633–031A2, TLC Pharmaceutical Standards). The lower limit of quantification (LLOQ) of delafloxacin was 0.04 µg/mL.

### Non-compartmental pharmacokinetic analysis

The pharmacokinetic parameters, including maximum concentration (C_max_), area under the curve (AUC), the CL, and volume of distribution (V_d_), were estimated using the non-compartmental model with Phoenix WinNonlin (version 8.3). For the single 300 mg dose group, a bioequivalence analysis was conducted between the test drug and the reference drug. The test drug was an injection of delafloxacin developed and produced by Jiangsu Aosaikang Pharmaceutical Co. Ltd., and the reference drug was an injection of delafloxacin produced by Melinta Therapeutics Inc. The pharmacokinetic characteristics of the two formulations, including C_max_, AUC_0-∞_, and AUC_0-t_, were compared, and their bioequivalence was assessed. The descriptive and inferential statistical analyses were conducted using the R language (version 4.2.1).

### Population pharmacokinetic analysis

The non-linear mixed-effects modeling software NONMEM (version 7.5, ICON Development Solutions, MD, United States) was used to construct the PopPK model. The first-order conditional estimation with interaction (FOCE-I) method was chosen to estimate the fixed effect parameter of the model. As the percentage of below quantification limit (BQL) data was less than 10%, BQL data were removed (M1 method). Also, Perl-speaks-NONMEM (PsN, version 5.2.6, Uppsala University, Sweden) and Pirana (version 3.0.0, Certara, United States) were employed for processing model outcomes and data. The R package tidyverse (version 1.3.2) and tidyvpc (version 1.4.0, Certara, United States) were utilized for data analysis, visualization, and plotting visual predictive check (VPC).

### Base model development

The data from both the single-dose and multiple-dose groups were incorporated into a PopPK model. Based on the results of exploratory analysis and published literature (14, 15), one-compartment, two-compartment, and three-compartment models were established and that performing the best was selected to serve as the base model. Inter-individual variability of PK parameters was characterized by the exponential error model equation.1


(Eq.1) $$P_{i}=\theta *exp(\eta_{i})$$

Where $P_{i}$ is the individual value of the parameter, θ is the typical population value of the parameter, and $\eta_{i}$ represents a series of normally distributed random numbers with a mean of 0 and a variance of $\omega^{2}$ .

The residual error structure was evaluated with combined error models. The equations are as follows equation. 2


(Eq.2) $$Y=IPRED\times (1+\varepsilon_{2})+\varepsilon_{1}$$

Where Y is the observed value for each individual and IPRED is the predicted value for each individual, $\varepsilon_{1}$ is the additive error, and $\varepsilon_{2}$ is the proportional error with a mean of 0 and a variance of $\sigma_{1}^{2}$ and $\sigma_{2}^{2}$ .

### Covariate analysis

Subsequently, correlations between covariates were investigated using both exploratory and statistical analyses. Categorical covariates underwent Analyses of Variance (ANOVA) tests, whereas continuous covariates were analyzed using linear regression. Among the highly correlated covariates, the most biologically plausible covariates were selected for stepwise covariate modeling (forward: *P* < 0.05; backward: *P* < 0.01). Continuous covariates were introduced into the model using a power approach, and categorical covariates into the model using a linear approach equations 3 and 4.


(Eq.3) $$P_{i}=\theta_{1}∙{\left(\frac{{COV}_{i}}{{COV}_{median}}\right)}^{\theta_{2}}$$

(Eq.4) $$P_{i}=\theta_{1}\cdot \left(1+\theta_{2}\cdot COV_{cat,i}\right)$$

Where $\theta_{1}$ is the mean value of the parameter among individuals with median covariate values, $\theta_{2}$ represents the estimated typical value of the covariate’s effect on $\theta_{1}$ . ${COV}_{i}$ is the continuous covariate value for the i^th^ individual, whereas ${COV}_{cat,i}$ is the categorical covariate value for the i^th^ individual. ${COV}_{median}$ is the median value of continuous covariates, and $P_{i}$ represents the parameter for the i^th^ individual adjusted for covariates.

For continuous covariates, specific values were derived from the distribution of included covariates, namely the 5^th^, 25^th^, 50^th^ (median, used as the reference value), 75^th^, and 95^th^ percentiles. These values were employed to calculate the influence of covariates on pharmacokinetic parameters. For categorical covariates, all categories present in the data set were considered. Each process of one changed covariate was repeated 1000 times and was identified with R package coveffectsplot (version 1.0.4) (18).

### Model validation

The final model was validated primarily using Goodness-of-Fit (GOF) plots, bootstrap analysis, and VPC. In the bootstrap analysis, 1,000 sets of parameters were estimated by stratifying the original data and repeatedly sampling 1,000 data sets. The 95% confidence intervals (95% CI) for model parameters were constructed based on bootstrap results. VPC was employed to assess the predictive ability of the final model. This assessment involved simulating the changes in blood concentrations over time and comparing the results of 1,000 simulations with the original data to evaluate the model’s predictive performance.

### Dose optimization via Monte Carlo simulations

According to previous research (17), delafloxacin exerted its bactericidal effect in a concentration-dependent manner. Thus, *f*AUC/MIC (a free fraction of 0.16) was considered as the PK/PD parameter that best predicted the efficacy of clinically approved fluoroquinolones. The probability of target attainment (PTA) for delafloxacin, associated with a 1-log10 CFU reduction, represented the percentage of PK/PD parameters achieving the target value at a specific MIC level. The cumulative fraction of response (CFR) was calculated by weighing the PTAs corresponding to different MICs, based on the proportion of each MIC within the population.

Monte Carlo simulations were conducted by NONMEM to assess the PK profiles of delafloxacin at doses of 150, 200, 300, 450, and 600 mg. As indicated on its label, delafloxacin (Baxdela) is recommended to be dosed at 300 mg q12h. Therefore, we simulated the PK profiles in 1,000 virtual patients at each dose. The target *f*AUC/MIC values, based on preclinical pharmacodynamic targets, were set at 14.3, 24.7, and 31.8 for *S. aureus*, MRSA, *Streptococcus pneumoniae*, respectively (16, 19). The PK/PD target attainment analysis was used to determine the optimal doses for different populations. Monte Carlo simulations were performed based on NONMEM, and visual analysis was performed using The R package tidyverse.

## RESULTS

### Subject characteristics

The demographic characteristics of study subjects are summarized in Table 1, whereas the remaining details are shown in Table S1. During the clinical trial, one subject in the 300 mg single-dose group withdrew from the first cycle due to concomitant medication, whereas another in the 600 mg single-dose group withdrew due to a vasovagal reaction during drug administration. A total of 58 subjects were included in the PK concentration set (PKCS).

**TABLE 1:** Demographic information of the study population

Variables Median [min, max] (n%)	Single dose			Multiple dose	Overall
	150 mg	300 mg	600 mg	300 mg	
	(*N* = 12)	(*N* = 23)	(*N* = 11)	(*N* = 12)	(*N* = 58)
Age (years)	25.0 [20.0, 34.0]	31.0 [21.0, 43.0]	28.0 [18.0, 43.0]	25.0 [20.0, 35.0]	28.0 [18.0, 43.0]
Weight (kg)	66.7 [46.9, 81.2]	59.3 [45.0, 77.9]	60.9 [46.7, 70.8]	60.0 [47.3, 75.5]	61.9 [45.0, 81.2]
Height (cm)	169 [154, 185]	166 [146, 183]	168 [150, 174]	165 [154, 176]	168 [146, 185]
BMI (kg/m2)	23.9 [19.3, 24.9]	22.9 [19.8, 25.7]	22.5 [19.2, 25.2]	23.0 [19.4, 24.4]	23.0 [19.2, 25.7]
Sex					
Female	5 (41.7%)	11 (47.8%)	4 (36.4%)	7 (58.3%)	27 (46.6%)
Male	7 (58.3%)	12 (52.2%)	7 (63.6%)	5 (41.7%)	31 (53.4%)
Renal creatinine clearance (mL/min)	129 [105, 148]	128 [85.0, 173]	120 [100, 143]	125 [110, 164]	126 [85.0, 173]
Serum creatinine (μmol/L)	70.5 [40.0, 100]	58.0 [43.0, 80.0]	68.0 [47.0, 86.0]	57.5 [47.0, 84.0]	62.0 [40.0, 100]
eGFR (mL/min/1.73 m^2^)	120 [85.7, 215]	137 [108, 189]	133 [93.5, 166]	138 [102, 172]	135 [85.7, 215]

^*a*^ n%, percentage of subjects; BMI, body mass index; eGFR, estimated glomerular function rate. eGFR was calculated with modified MDRD formula for Chinese and renal creatinine clearance was calculated according to the Cockcroft–Gault (CG) formula.

### Safety of delafloxacin

Healthy Chinese subjects tolerated single doses of 150 mg, 300 mg, and 600 mg, as well as multiple doses of 300 mg intravenous delafloxacin, indicating its high safety. There were no significant differences in safety between the test and reference drugs administered by intravenous infusion.

### PK characteristics of delafloxacin

**Fig 1:**
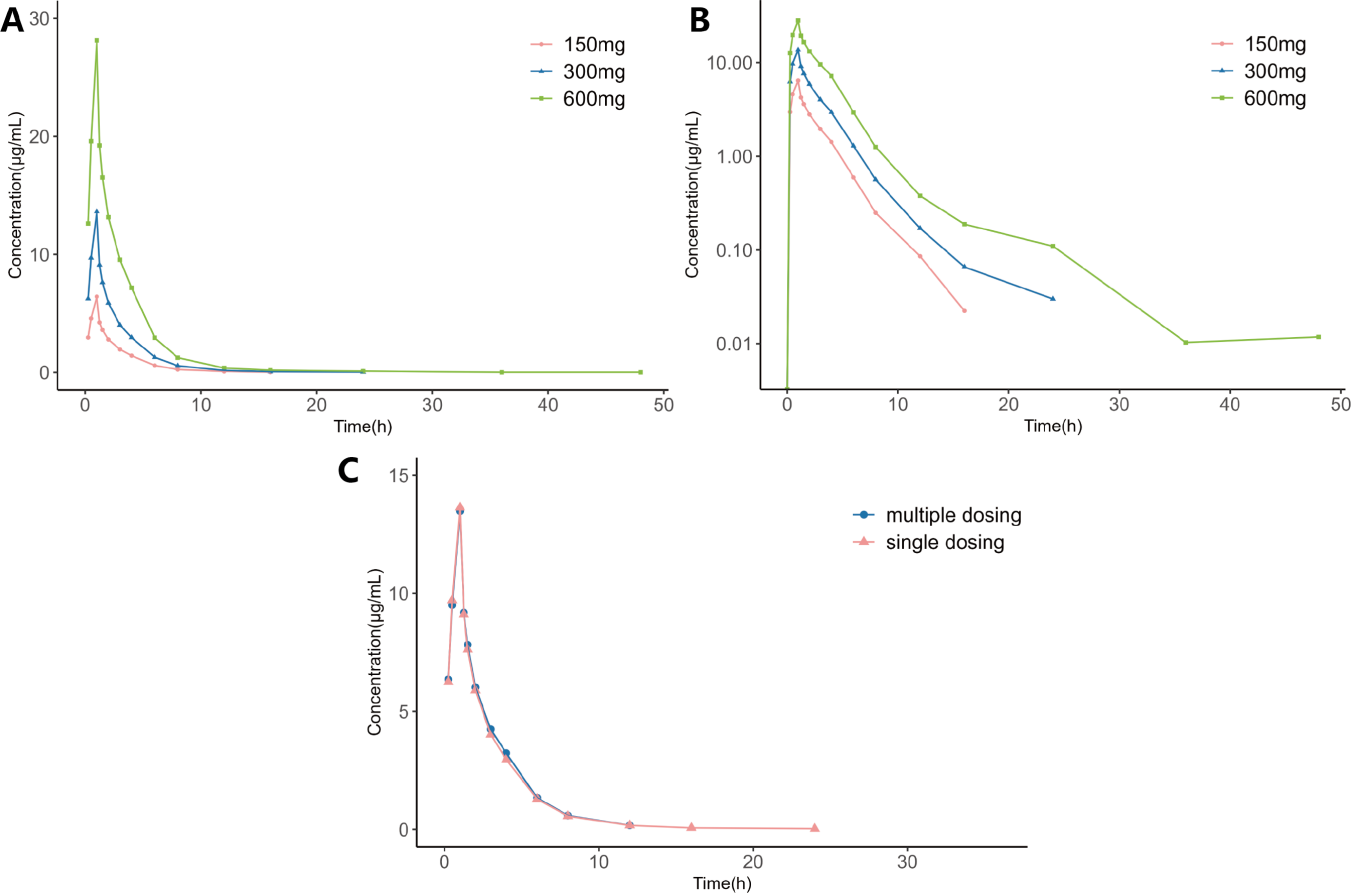
Mean plasma concentration-time profile of delafloxacin in healthy Chinese subjects. (**A**) Mean plasma concentration-time profile after one intravenous injection of delafloxacin. (**B**) Semi-log plot of plasma concentration-time profile after one intravenous injection of delafloxacin. (**C**) Mean plasma concentration-time profile of delafloxacin after one and multiple intravenous injections of 300 mg.

The concentration-time profiles for each dose group and semi-log concentration-time plots are illustrated in Fig. 1. Dose-normalized geometric mean of concentration time is shown in Fig. S1. The PK parameters for all dose groups based on the noncompartmental analysis are summarized in Table S1.

In our study involving Chinese healthy subjects, there were no statistical sex-related differences in terms of t_1/2_, T_max_, C_max_, AUC_0-t_, AUC_0-∞_, CL, and Vd in both the single-dose 150 mg or 300 mg groups. All of PK parameters were based on noncompartmental analysis. In both the single-dose 600 mg and multiple-dose 300 mg groups, there were no statistical sex-related differences in C_max_, AUC_0-t_, AUC_0-∞_, t_1/2_, CL, Vd, and T_max_.

AUC_0-t_ increased with dose in various single-dose groups. Linear PK analysis demonstrated that the slopes of plasma delafloxacin C_max_, AUC_0-t_, and AUC_0-∞_ were 1.07 (0.99, 1.16, 90% CI), 1.13 (1.04, 1.23, 90% CI), and 1.13 (1.04, 1.22, 90% CI), respectively. The linear relationship analysis is presented in Table S2. PK results from the multiple-dose group showed that a steady state of plasma delafloxacin was achieved at day 6, with minimal accumulation.

Furthermore, a bioequivalence study was conducted, and two-sided *t*-tests confirmed the bioequivalence between the text and the reference drugs, suggesting that the delafloxacin injections produced by the two companies were bioequivalent in healthy Chinese subjects.

### Delafloxacin PopPK modeling

**Fig 2:**
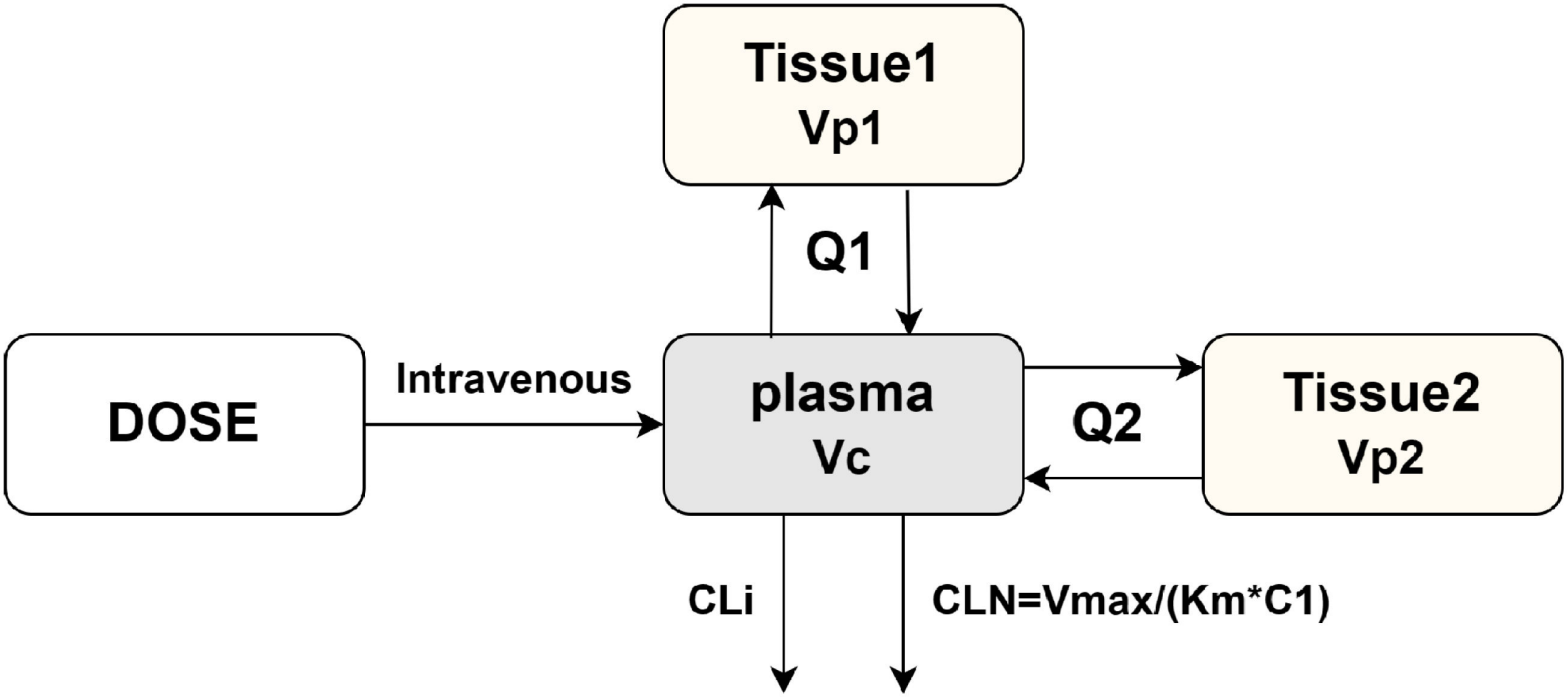
Schematic structure of the final PopPK model for delafloxacin. Vc, the central volume; Vp, the peripheral volume; CL = total clearance (calculated as CLN +CLi); CLi = linear clearance from the central compartment; CLN = nonlinear clearance from the central compartment (calculated as Vmax*KM/(KM +C1)); C1, concentration of the drug in the central compartment.


For delafloxacin, a 3-compartment model with mixed linear and nonlinear CL (objective function value, OFV = −870) could provide the best fit to the data, compared with two-compartment model (OFV = −682) and three-compartment model (OFV = −764) with liner CL. Hence, we chose a three-compartment model with parallel linear and nonlinear CL for PopPK modeling (Fig. 2). Total CL of the delafloxacin involved CL via the nonlinear pathway (CLN) plus that via the linear pathway (CLi). CLN was described by a Michaelis-Menten equation. 5.

(Eq.5) CLN=Vmax/(KM+C1)

Where Vmax is the maximum elimination rate, KM is the concentration at which elimination is at half maximum, and C1 is the concentration of the drug in the central compartment.

**Fig 3:**
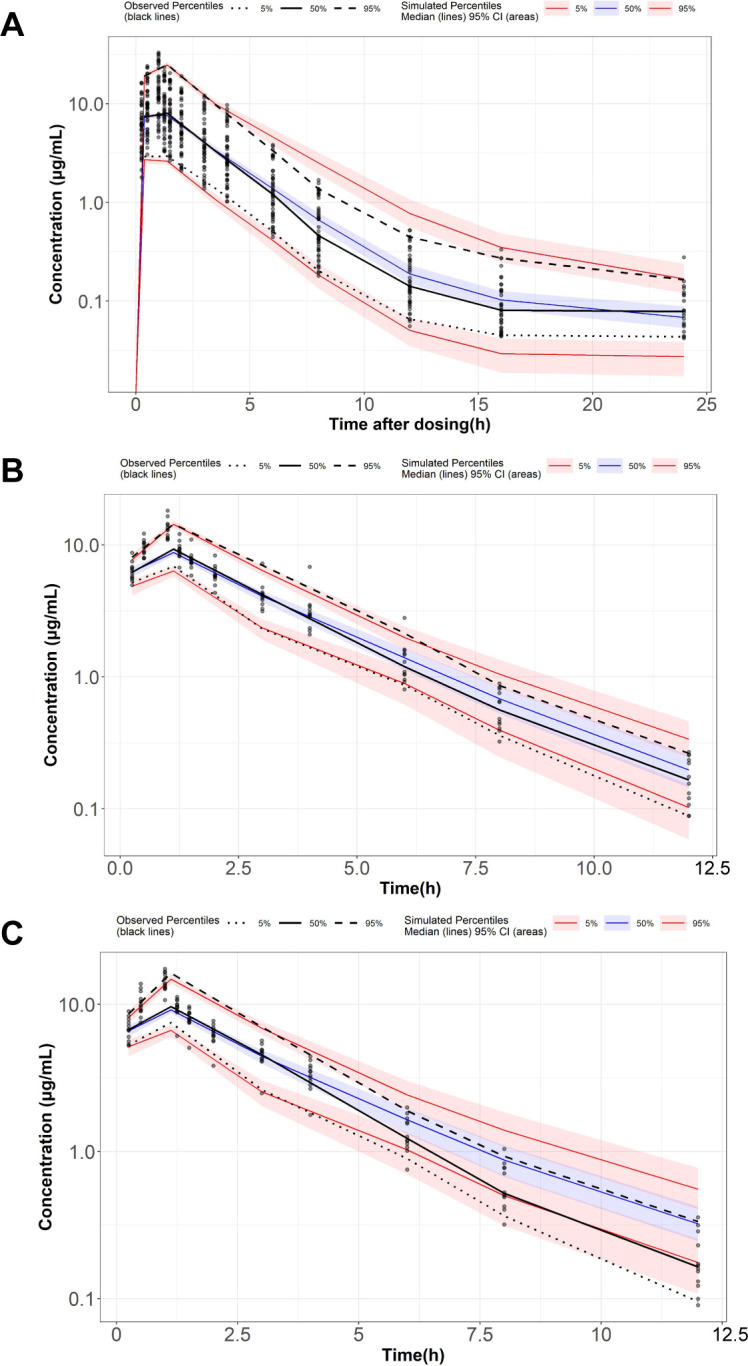
Prediction-corrected visual predictive check of the final PopPK model for delafloxacin. (**A**) Semi-log plot of single ascending dose study. (**B**) Semi-log plot of multiple ascending dose study on day 1. (**C**) Semi-log plot of multiple ascending dose study on day 5. Circles represent observed data. Black lines represent the 5% (dashed), 50% (solid), and 95% (dashed) percentiles of the observed data. Shaded areas represent 95% confidence intervals of the median 5% (red), 50% (blue), and 95% (red) percentiles of the predicted concentrations. For A and B, the red and black lines represent the median of predicted and observed BQL fractions, respectively. Shaded areas represent 90% prediction intervals.

The final delafloxacin model is presented in the supplemental material. All parameters were estimated with an RSE of <40% in the final valsartan model, demonstrating an acceptable precision (Table 2). The bootstrap results indicated that all estimates were within the 95% confidence intervals obtained from 1,000 bootstrap runs, suggesting the stability of the final model. Additionally, the goodness-of-fit plots (Fig. S2) confirmed a good fit, and prediction-corrected visual predictive check (VPC) plots (Fig. 3) showed a strong predictive performance of the final model.

**TABLE 2:** Parameter estimates of the final naloxone population pharmacokinetic model and bootstrap results

Delafloxacin final model			Bootstrap Median [95% CI]
Fixed effect parameters	Final estimation	RSE	
CL, L/h	4.54	3.6%	4.56 [4.24–4.83]
V1, L	7.36	3.3%	7.34 [6.94–7.78]
V2, L	15.0	2.7%	14.97 [14.27–15.66]
V3, L	18.1	6.5%	17.80 [12.39–23.75]
Q2, L/h	25.8	3.1%	25.87 [24.40–27.20]
Q3, L/h	0.96	6.1%	0.96 [0.84–1.07]
VM, L/h	40	FIX	NA
KM, μg/mL	5	FIX	NA
The effect of weight on CL	1.13	19.8%	1.13 [0.76–1.50]
The effect of weight on V1	1.38	15.0%	1.38 [1.01–1.74]
Interindividual variability (%CV)	Final estimation	RSE[Shrinkage]	Bootstrap Median [95% CI]
IIV_CL	0.066	17% [2.4%]	0.063 [0.048–0.084]
IIV_V1	0.041	20.9% [9.2%]	0.039 [0.026–0.084]
IIV_V2	0.027	19.6% [8%]	0.027 [0.018–0.036]
IIV_Q3	0.1	FIX	NA
Residual unexplained error (%CV)	Final estimation	RSE	Bootstrap Median [95% CI]
prop.err (CV%)	0.073	9.8%	0.073 [0.06–0.08]
add.err (CV%)	0.1	13.9%	0.1 [0.07–0.13]

^*a*^ %RSE, percent relative standard error; CI, confidence interval; %CV, coefficient of variation; KM, concentration at which elimination is at half maximum (the Michaelis-Menten constant); VM, maximum elimination rate: NA, not applicable.

**Fig 4:**
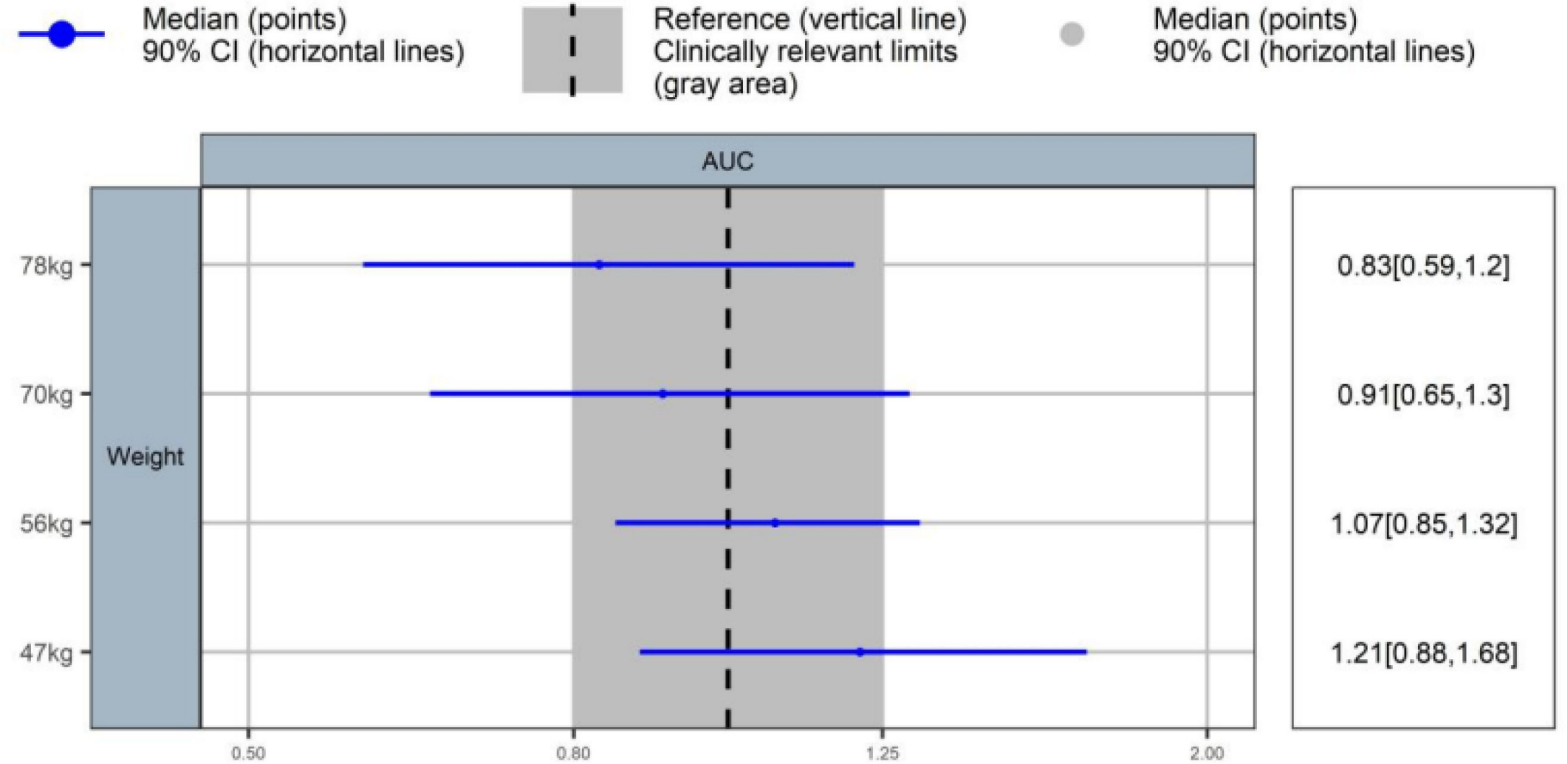
The impact of covariates on the steady state exposure of delafloxacin. The demographic characteristics of simulated reference subjects were: weight of 62 kg.

After covariate correlation analysis and the SCM procedure, one covariate (weight) was retained in the final PK model. The code of the SCM procedure is presented in the supplemental material. The correlations between continuous and categorical covariates are shown in Fig. S3. The influence of selected covariates on the typical pharmacokinetic profiles of delafloxacin is depicted in the forest plot (Fig. 4). There was a positive relationship between age and AUC_ss,24h_. Compared with a 62 kg patient (median), a 47 kg patient (5^th^ percentile) and a 78 kg (95^th^ percentile) patient experienced an increase and decrease of approximately 20% in AUC_ss,24h_.

### Monte Carlo simulation and PK/PD analysis

**Fig 5:**
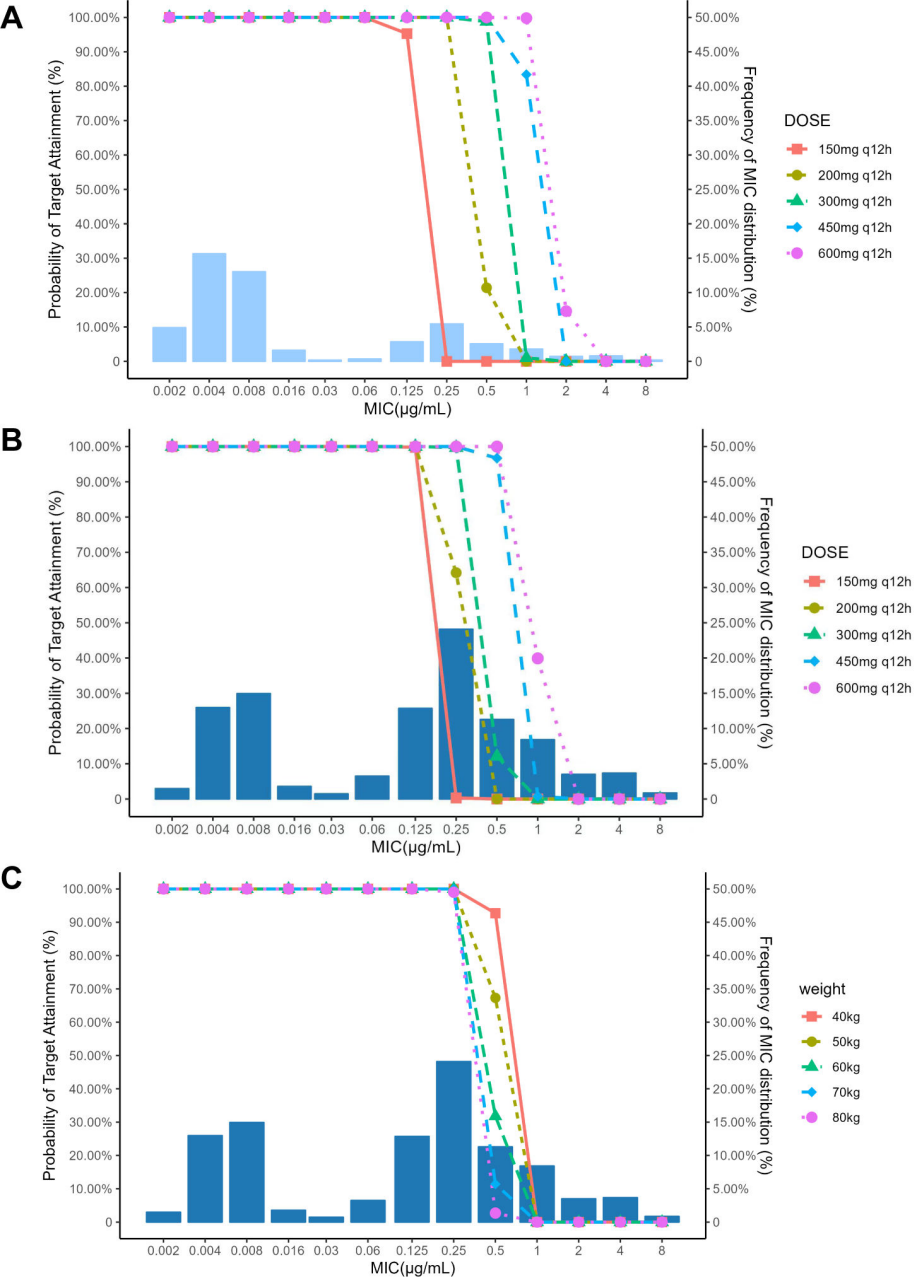
(**A**) PTA results of different doses of delafloxacin against *S. aureus*. (**B**) PTA results of different doses of delafloxacin against MRSA. (**C**) PTA results of delafloxacin against MRSA in patients with different weights after administering 300 mg. The lines show the calculated PTA of delafloxacin at each simulated dose, and the bar chart is the distribution of MIC.

After model analysis and covariate correlation analysis, five doses were administered in a simulated population, including 150 mg q12h, 200 mg q12h, 300 mg q12h, 450 mg q12h, and 600 mg q12h. The simulated population consisted of patients with a weight of 70 kg. According to *in vitro* study in literature (7, 16, 17, 20–22), the common MIC range for delafloxacin is 0.008–4, with a free fraction of 0.16. The PTA values for different *f*AUC/MIC targets of *S. aureus* including MRSA under various dose regimens at steady state are shown in Fig. 5A and B. Most *S. aureus*, including MRSA strains, exhibited MICs lower than 0.25 µg/mL, and MRSA strains presented the highest at MIC = 0.25 µg/mL. Also, report (21) from a phase III clinical trial indicates that the MIC_90_ value for MRSA in Asian and Latin American populations is 0.25 µg/mL. Additionally, MIC statistics from Europe also support the rationale for selecting 0.25 µg/mL as the susceptibility breakpoint for MIC_90_. The PTA of all dosage groups was >90% against *S. aureus* with an MIC ≤0.25 µg/mL. Furthermore, both the 200 mg and 150 mg dose groups exhibited CFR values below 90% against *S. aureus*, whereas all the other dose groups exhibited higher CFR values. The PTA of 200 mg q12h against MRSA was only 64.2% with an MIC = 0.25 µg/mL, whereas that of 300 mg q12h was >90%.

**Fig 6:**
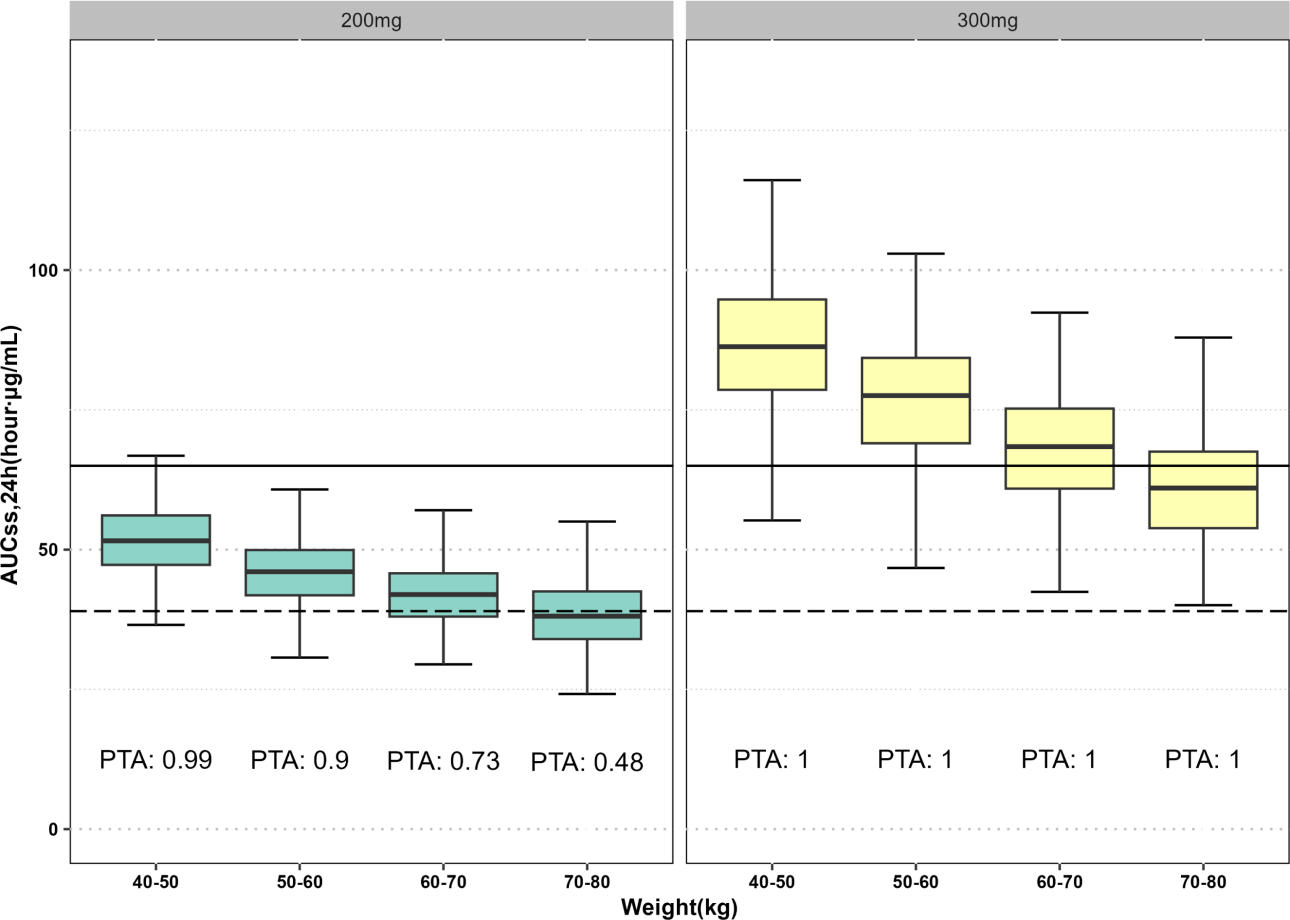
Simulation of intravenous delafloxacin steady state exposure in ABSSSI patients caused by MRSA at different weights and doses. The dashed line is the AUC_ss,24th_ values required to achieve the target *f*AUC/MIC values (24.7) associated with a 1-log10 CFU reduction at MIC = 0.25 µg/mL (MIC_90_), which is effective against MRSA, and the solid line is the simulated average AUC_ss,24th_ value after administering 300 mg to individuals weighing of 70 kg. The PTA for delafloxacin, associated with a 1-log10 CFU reduction, represented the percentage of PK/PD parameters achieving the target *f*AUC/MIC values (24.7) at MIC = 0.25 µg/mL (MIC_90_) on each box plot.

Our subjects’ weights ranged from 40 to 80 kg. Subsequently, we conducted simulations to assess the pharmacological effects following administration across various weight categories (40–50 kg, 50–60 kg, 60–70 kg, and 70–80 kg). In different weight groups, the PTA values of 300 mg q12h against MRSA are presented in Fig. 5C. When MIC was ≤0.25 µg/mL, the PTA of 300 mg q12h against MRSA was >90% in all weight groups. The simulated exposure levels of 200 mg and 300 mg in patients with different weights are shown in Fig. 6. In patients weighing less than 60 kg, a dose of 200 mg q12h achieved a favorable therapeutic effect against MRSA. The exposure levels in patients receiving a dose of 200 mg q12h were lower than those in patients receiving a dose of 300 mg q12h.

## DISCUSSION

To the best of our knowledge, this was the first PopPK model-based analysis to describe the PK/PD characteristics of delafloxacin in the Chinese population, as well as provide optimal dosages for different populations. Compared with those observed in phase I clinical trials in America, this study demonstrated higher average PK parameters (C_max_ and AUC). Notably, the t_max_ and half-life aligned with those indicated at the label of delafloxacin. The accumulation index after multiple doses was 1.47, indicating that the drug did not accumulate to a high level. Additionally, the bioequivalence study indicated that the test drug used in this trial was bioequivalent to the reference drug. The overall PK results of this study are consistent with those reported previously (5, 9, 23). The PK linear analysis result revealed that the CIs had a range of 0.84–1.16, indicating that C_max_ exhibited pharmacokinetic characteristics linear with dose, whereas the linearity of AUC_0-t_ and AUC_0-∞_ within a specified dose range was not definite. Consistent with findings from the study conducted in America (9), the slope of delafloxacin AUC fell completely outside the CI, indicating a lack of linearity between AUC in Chinese. The reasons for this unclear linear relationship remain unclear but may involve the saturation of elimination pathways. In contrast, the linear relationship of delafloxacin in the Chinese healthy population appears to be more typical, compared with that observed in other countries.

To better understand the PK/PD characteristics of delafloxacin in Chinese, a 3-compartment model with mixed linear and nonlinear CL was adopted. In this model, due to the presence of additional nonlinear elimination pathways, it is reasonable to consider that the CL is lower than that calculated in the non-compartmental analysis (NCA). Furthermore, in comparison to the PopPK models reported (14, 15), the estimated CL values in our study were slightly lower, which may be attributed to the fact that the subjects in America had heavier body weights compared with those in our research, whereas the CL rate increases with body weight.

Based on the FDA review of clinical trials (19) of delafloxacin, the PK characteristics of delafloxacin in both healthy individuals and patient populations are consistent. Therefore, we did not include disease-related factors as covariates in the PopPK model. In contrast to other models, our model did not incorporate sex and renal function as covariates of CL because our raw data lacked a population with renal insufficiency. Also, one study in healthy women (9) shows that the effect of sex was not significant on any of the PK parameters of delafloxacin. However, we still verified the impact of body weight on the modeling results, especially the AUC_ss,24th_. Therefore, we designed dosing simulations based on the results of covariate analysis, thereby determining an optimal dosage.

Since most *S. aureus* including MRSA strains exhibit MICs lower than 0.25 µg/mL and an MIC_90_ of 0.25 µg/mL(7, 17, 21), we deem that such PTAs are suitable to treat ABSSSIs. Additionally, some *in vitro* studies (16, 17) suggest that when *f*AUC/MIC reaches the target of a 1-log10 CFU reduction, it can result in good therapeutic effects against MRSA, Methicillin-sensitive *S.aureus* (MSSA), and other strains. Considering that our study primarily focuses on MRSA strains, we find selecting a 1-log10 CFU reduction to be reasonable. The PTA results associated with a 1-log10 CFU reduction suggested that a dosage of 300 mg is effective for treating ABSSSIs caused by MRSA, aligning with the recommended dosage provided on the label of delafloxacin for patients with normal renal function. Furthermore, the clinical trial revealed a favorable safety observed in the Chinese population dosed at 300 mg. Therefore, it is recommended to proceed with 300 mg q12h for subsequent clinical research.

Our simulations suggested that a dose of 300 mg q12h can meet the clinical requirements across all weight groups. The covariate correlation analysis showed that a smaller weight corresponds to a greater exposure (AUC_ss,24h_), suggesting that to enhance safety, decrease antibiotic resistance, and meanwhile ensure clinical efficacy, exposure (AUC_ss,24h_) should be reduced in patients with a lower body weight. Therefore, we simulated the efficacy of 200 mg q12h delafloxacin across different weight groups. In a different study (24), a dose of 200 mg is recommended for patients with renal impairment. Our study demonstrated that a dose of 200 mg q12h can be applied for patients with a body weight of less than 60 kg. For a patient weighing less than 60 kg, a dose of 200 mg q12h should be reasonable to achieve the PK/PD target with an MIC of 0.25 µg/mL. Also, simulation results revealed that the exposure level in these patients after 200 mg dosing is lower than that in patients weighing 70 kg after 300 mg dosing. In summary, for patients with lower body weight, 200 mg q12h may be recommended to protect against ABSSSIs and reduce adverse drug reactions, which should be validated in future studies.

This study has its own limitations. First, our clinical data are limited by a comparatively small number of patients. Second, our clinical data lack information on patients with impaired liver and kidney function, body weight of more than 80 kg. Third, a deeper understanding of *in vivo* delafloxacin metabolism is needed to explain its non-linear elimination. What’s more, our study focused on the efficacy of delafloxacin in the treatment of ABSSSIs and did not include other indications. We plan to collect more data from different populations for further PK/PD study of delafloxacin in Chinese.

### Conclusion

In conclusion, this was the first study to show that intravenous delafloxacin might present a high safety in Chinese. Additionally, its PK characteristics were consistent with those indicated on the label of delafloxacin. A new PopPK model for delafloxacin was developed to simulate the PK characteristics in the Chinese population. Considering that renal function was not included in the model, our simulated predictions pertain to patients with normal renal function. An optimal dose of 300 mg q12h was recommended to treat ABSSSIs caused by *S. aureus* even MRSA, with satisfactory efficacy and safety when the body weight was more than 60 kg. For those with a body weight of less than 60 kg, the dose of 200 mg could achieve a good therapeutic effect while reducing the incidence of adverse reactions.

## SUPPLEMENTAL MATERIAL

Supplemental material aac.00428-24-s0001.docx Supplemental methods and results, Tables S1 and S2, and Fig. S1 to S3.

ASM does not own the copyrights to Supplemental Material that may be linked to, or accessed through, an article. The authors have granted ASM a non-exclusive, world-wide license to publish the Supplemental Material files. Please contact the corresponding author directly for reuse.
