# Supplementary material for: Pharmacokinetics and pharmacodynamics of intravenous delafloxacin in healthy subjects: model-based dose optimization
Source: Antimicrob Agents Chemother. 2024 Jun 20;68(7):e00428-24. doi: 10.1128/aac.00428-24 (PMC11232397; doi:10.1128/aac.00428-24)
Supplement: Supplemental material — Supplemental methods and results, Tables S1 and S2, and Fig. S1 to S3. [file aac.00428-24-s0001.docx]

**SUPPLEMENTARY MATERIAL**

**Supplementary Methods**

Exclusion criteria

Exclusion criteria included:

1. Subjects with an allergy to the investigational drug or to other quinolone antibiotics.
2. Subjects with a pulse rate of <55 beats per minute or >100 beats per minute, and a body temperature (ear temperature) of <35.7℃ or >37.5℃.
3. Subjects with systolic blood pressure <90 mmHg or >140 mmHg, and diastolic blood pressure >90 mmHg or <60 mmHg.
4. Subjects with a history of cardiac, hepatic, renal, respiratory, gastrointestinal, or neurological disease, and a history of psychiatric or metabolic disease.
5. Subjects who have undergone major surgical procedures in the three months prior to screening, are planning to undergo surgery during the trial, or have had surgery that could affect the pharmacokinetic properties of the study drug.
6. Subjects with a history of smoking, alcohol consumption, or drug use.
7. Subjects who are pregnant, unable to comply with a standardized diet, or have received vaccinations within the 28 days prior to screening.

Delafloxacin dosing scheme

The single-dose trial consisted of three dose groups, 150 mg, 300 mg, and 600 mg, which were administered when subjects fasted for at least 10 hours overnight. The multiple-dose trial only had one dose group, 300 mg, and subjects fasted for a minimum of 10 hours overnight on the first and fifth days. They received a 300 mg dose of delafloxacin via intravenous infusion in the evening of the first day, and 300 mg doses in the morning and evening from the second to the fifth day. On the sixth day, they took the morning dose. The dosing intervals were 12 hours.

**Supplementary Results**

The code of final delafloxacin model

$INPUT C STUDYID ID DOSE TIME DV AMT CMT RATE ADDL II EVID MDV BQL SEX WT HT BMI AGE CCR eGFR LDL HDL LDH ALT AST ALB TP CREA WBC RBC HGB PLT NEUT PT PTA PH

$DATA DLSX8-5.csv IGNORE=C

$SUBROUTINES ADVAN6 TOL=4

$MODEL

COMP=(CENTRAL)

COMP=(PERIPH1)

COMP=(PERIPH2)

V1WT = ((WT/61.9)**THETA(12))

V1COV=V1WT

CLWT = ((WT/61.9)**THETA(11))

CLCOV=CLWT

TVCL=THETA(1)

TVCL = CLCOV*TVCL

CL=TVCL * EXP(ETA(1))

TVV1=THETA(2)

TVV1 = V1COV*TVV1

V1= TVV1* EXP(ETA(2))

TVV2=THETA(3)

V2=TVV2 * EXP(ETA(3))

TVV3=THETA(4)

V3= TVV3* EXP(ETA(4))

TVQ2=THETA(5)

Q2=TVQ2* EXP(ETA(5))

TVQ3=THETA(6)

Q3= TVQ3* EXP(ETA(6))

TVVM=THETA(7)

VM= TVVM*EXP(ETA(7))

TVKM=THETA(8)

KM= TVKM*EXP(ETA(8))

K10=CL/V1

K12=Q2/V1

K21=Q2/V2

K13=Q3/V1

K31=Q3/V3

S1=V1

S2=V2

S3=V3

$DES

DADT(1)= K21*A(2)+K31*A(3)-(K12+K13+K10)*A(1)-VM*A(1)/(KM*V1+A(1))

DADT(2)= K12*A(1)-K21*A(2)

DADT(3)= K13*A(1)-K31*A(3)

$ERROR

IPRED = F

W = SQRT(THETA(9)**2*IPRED**2 + THETA(10)**2)

Y = IPRED + W*EPS(1)

IRES = DV-IPRED

IWRES = IRES/W

$THETA (0,4.54924) ; CL

(0,7.3707) ; V1

(0,16.9656) ; V2

(0,17.8772) ; V3

(0,25.7329) ; Q2

(0,0.944349) ; Q3

40 FIX ; VM

5 FIX ; KM

(0,0.0738488) ; prop.err

(0,0.104218) ; add.err

$THETA (-100,1.13272,100) ; CLWT1

$THETA (-100,1.36446,100) ; V1WT1

$OMEGA

0.0651272 ; IIV_CL

0.0415318 ; IIV_V1

0.0082784 ; IIV_V2

0 FIX ; IIV_V3

0 FIX ; IIV_Q2

0.1 FIX ; IIV_Q3

0 FIX ; IIV_KM

0 FIX ; IIV_VM

$SIGMA

1 FIX

$EST METHOD=1 INTERACTION MAXEVAL=1000 NOABORT NSIG=3 SIGL=9 PRINT=10

$COV UNCONDITIONAL SLOW

Covariate Evaluation

threads =40

search_direction=both

p_forward=0.05

p_backward=0.001

abort_on_fail=0

do_not_drop= HT BMI AGE CREA LDL AST ALB HGB WBC RBC TP PLT PT PH

continuous_covariates=WT,CCR,eGFR,LDH,HDL,ALT,NEUT,PTA

categorical_covariates=SEX

[test_relations]

CL=WT,eGFR,LDH,NEUT,PTA,SEX

V1=WT,eGFR,SEX

V2=HDL,ALT,NEUT,SEX

**Supplementary Tables**

TableS1. The demographic information of the study population.

| Variables  Median [min, max]  (n%) | Single dose | | | Multiple dose | Overall  (N=58) |
| --- | --- | --- | --- | --- | --- |
|  | 150mg | 300mg | 600mg | 300mg |  |
|  | (N=12) | (N=23) | (N=11) | (N=12) |  |
| white blood cells (10^9/L) | 6.00 [5.03, 8.63] | 6.07 [4.07, 9.14] | 6.38 [4.80, 7.64] | 5.75 [3.42, 8.78] | 6.05 [3.42, 9.14] |
| red blood cells (10^12/L) | 5.05 [3.91, 5.67] | 4.57 [3.78, 5.84] | 4.80 [3.87, 5.50] | 4.53 [4.16, 5.52] | 4.63 [3.78, 5.84] |
| ALT (U/L) | 15.5 [8.00, 27.0] | 14.0 [7.00, 34.0] | 13.0 [9.00, 44.0] | 9.50 [8.00, 47.0] | 13.5 [7.00, 47.0] |
| ALB (g/L) | 49.6 [47.0, 52.4] | 48.0 [41.2, 51.1] | 49.4 [44.6, 52.7] | 49.4 [44.9, 54.8] | 49.1 [41.2, 54.8] |
| AST (U/L) | 18.5 [14.0, 30.0] | 16.0 [10.0, 22.0] | 14.0 [11.0, 26.0] | 15.0 [9.00, 22.0] | 15.5 [9.00, 30.0] |
| LDL (mmol/L) | 2.61 [2.03, 3.58] | 2.27 [1.46, 3.27] | 2.49 [1.16, 3.62] | 2.51 [1.02, 3.51] | 2.49 [1.02, 3.62] |
| LDH (U/L) | 160 [114, 190] | 147 [122, 184] | 159 [136, 204] | 158 [133, 230] | 155 [114, 230] |
| HDL (mmol/L) | 1.25 [0.950, 2.21] | 1.30 [1.03, 1.71] | 1.40 [0.940, 2.51] | 1.44 [1.05, 2.03] | 1.34 [0.940, 2.51] |
| Total protein (g/L) | 73.6 [66.9, 77.6] | 72.2 [61.2, 79.1] | 74.7 [69.4, 79.3] | 76.4 [69.1, 77.9] | 73.1 [61.2, 79.3] |
| Hemoglobin (g/L) | 155 [110, 174] | 143 [123, 177] | 151 [120, 168] | 135 [113, 162] | 146 [110, 177] |
| Platelets (10^9/L) | 265 [169, 436] | 266 [190, 355] | 301 [216, 336] | 240 [164, 337] | 266 [164, 436] |
| Neutrophil ratio (%) | 3.19 [2.86, 4.81] | 3.25 [1.96, 6.03] | 3.84 [2.28, 5.27] | 3.61 [1.71, 5.59] | 3.43 [1.71, 6.03] |
| Prothrombin time (sec) | 11.7 [10.6, 12.5] | 11.6 [10.4, 13.5] | 11.2 [10.0, 12.4] | 11.2 [10.1, 13.0] | 11.6 [10.0, 13.5] |
| Prothrombin activity (%) | 88.0 [78.0, 103] | 89.0 [70.0, 107] | 94.0 [79.0, 115] | 95.0 [75.0, 112] | 89.0 [70.0, 115] |
| Urine pH | 6.00 [5.50, 7.00] | 6.00 [5.00, 7.00] | 6.00 [5.50, 6.50] | 6.25 [5.50, 7.00] | 6.00 [5.00, 7.00] |

Abbreviations: n%: percentage of subjects; ALT, alanine aminotransferase; AST, aspartate aminotransferase; LDL, low-density lipoprotein; HDL, high-density lipoprotein; LDH, lactic dehydrogenase.

Table S2. Plasma pharmacokinetic parameters in healthy Chinese subjects after a single and multiple intravenous infusion of delafloxacin

| Pharmacokinetic  parameter | Delafloxacin single dose | | | Delafloxacin multiple dose |
| --- | --- | --- | --- | --- |
|  | 150mg  (N=12) | 300mg  (N=23) | 600mg  (N=11) | 300mg  (N=12) |
| T_max_(hour) | 0.96(0.92,1.03) | 0.96(0.90,0.98) | 0.96(0.90,1.02) | 0.94(0.90,1.02) |
| C_max_(μg/mL) | 6.43(1.14) | 13.63(2.57) | 28.14(3.10) | 14.29(2.00) |
| AUC_0-t_(hour**∙**μg/mL) | 15.71(2.71) | 33.69(7.34) | 75.23(10.12) | NA |
| AUC_0-τ,ss_(hour**∙**μg/mL) | NA | NA | NA | 35.44(5.23) |
| T_1/2_(hour) | 2.35(0.75) | 3.41(3.70) | 5.21(4.43) | 6.84(6.64) |
| CL(mL/hour) | 9681.55(1643.57) | 9208.76(1875.42) | 8033.62(1101.45) | 8676.42(1593.63) |
| Vd(mL) | 32719.42(10780.90) | 45071.43(47943.06) | 60356.43(53194.56) | 25210.70(6839.74) |
| R | NA | NA | NA | 1.48(0.73) |

Abbreviations: T_max_, The time point corresponding to the maximal concentration; C_max_, maximal concentration; AUC_0-t_, area under the concentration–time curve from time 0 to the last quantifiable concentration; AUC_0-τ,ss_, area under the steady-state concentration–time curve from from the last dose to the dosing interval; T_½_, half-life; CL, clearance; Vd, volume of distribution; R, accumulation index.

Table S2. Linear relationship analysis of PK parameters for the different single-dose groups

| Pharmacokinetic parameter | Slope | P value | The 90% confidence interval | The reference interval |
| --- | --- | --- | --- | --- |
| C_max_(µg/mL) | 1.07 | <0.001 | (0.99, 1.16) | (0.84, 1.16) |
| AUC_0-t_(hour∙µg /mL) | 1.13 | <0.001 | (1.04, 1.23) | (0.84, 1.16) |
| AUC_0-∞_(hour∙µg /mL) | 1.13 | <0.001 | (1.04, 1.22) | (0.84, 1.16) |

## Supplementary Figures

##
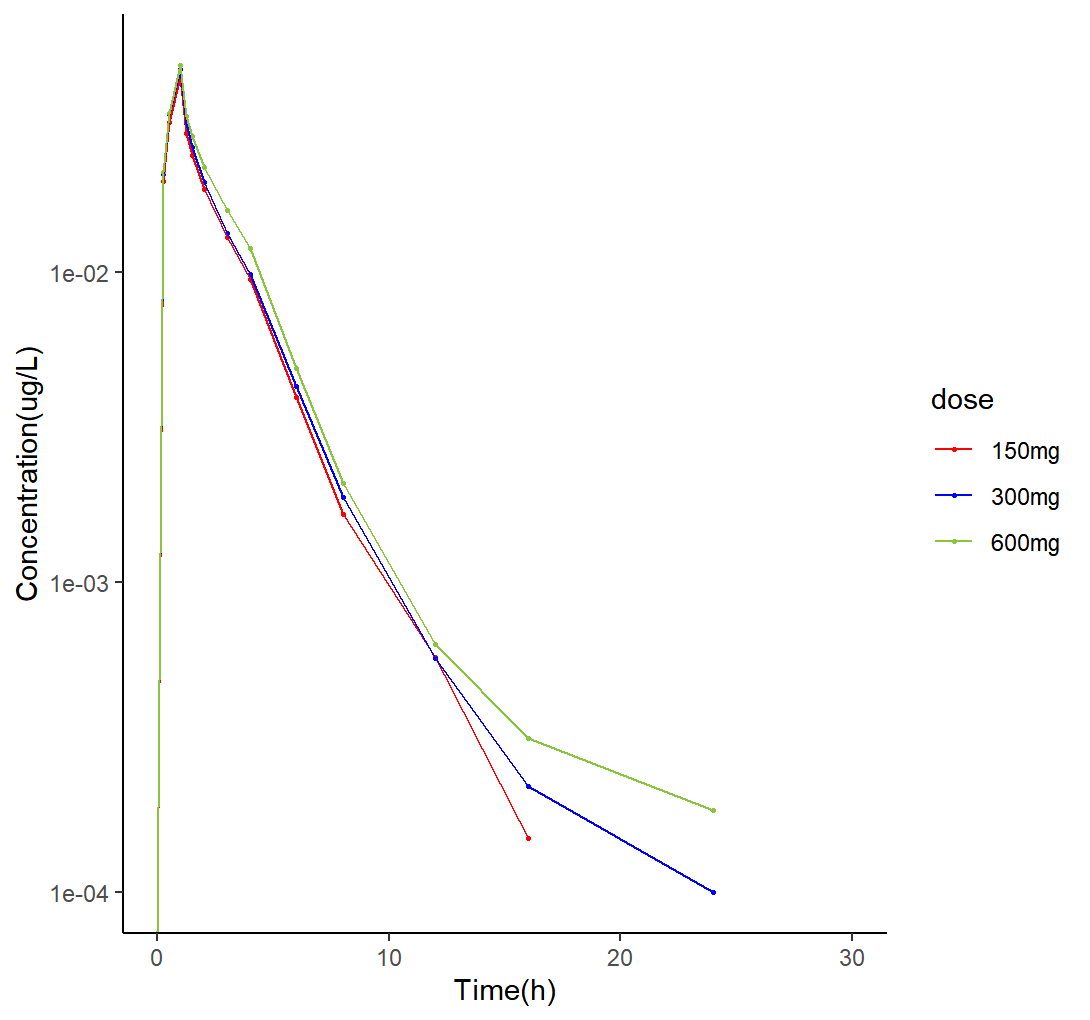


## Figure S1. Dose normalized geometric mean of concentration-time graph for single dose of delafloxacin . The scale of y axis is log and of x is normal.

##

##
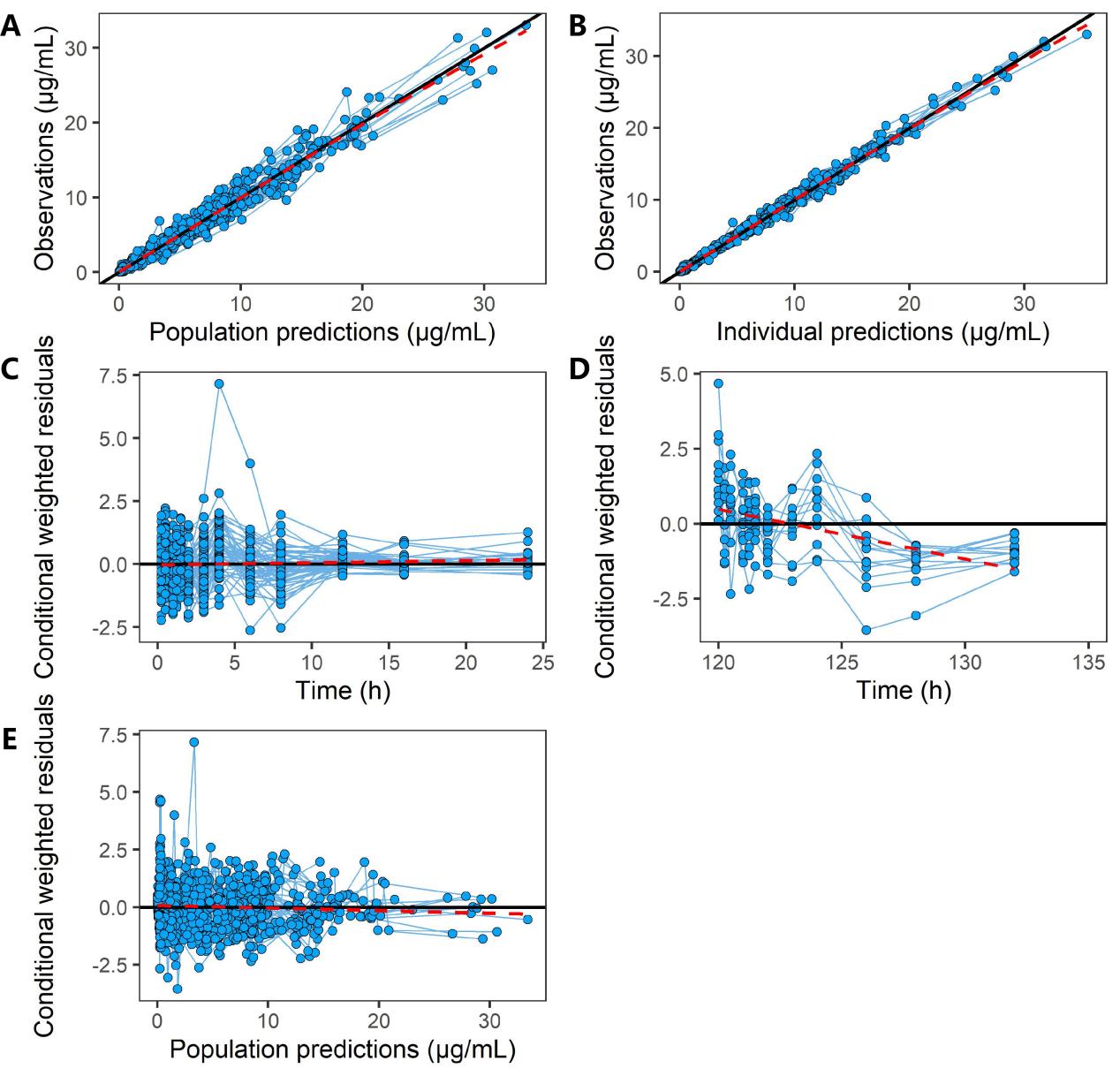


## Figure S2.**^1^** Goodness of fit for the final pharmacokinetic model of delafloxacin. The red line indicates the linear regression trendline and the black solid line means the reference line. (A) Light blue circles represent observed data. Plot of observed concentrations versus population predictions; (B) Plot of observations versus individual predictions. (C) Conditional weighted residual versus population predictions; (D) Conditional weighted residual versus time. The blue circles show observations. The red line shows smooth fitting for observations.

##
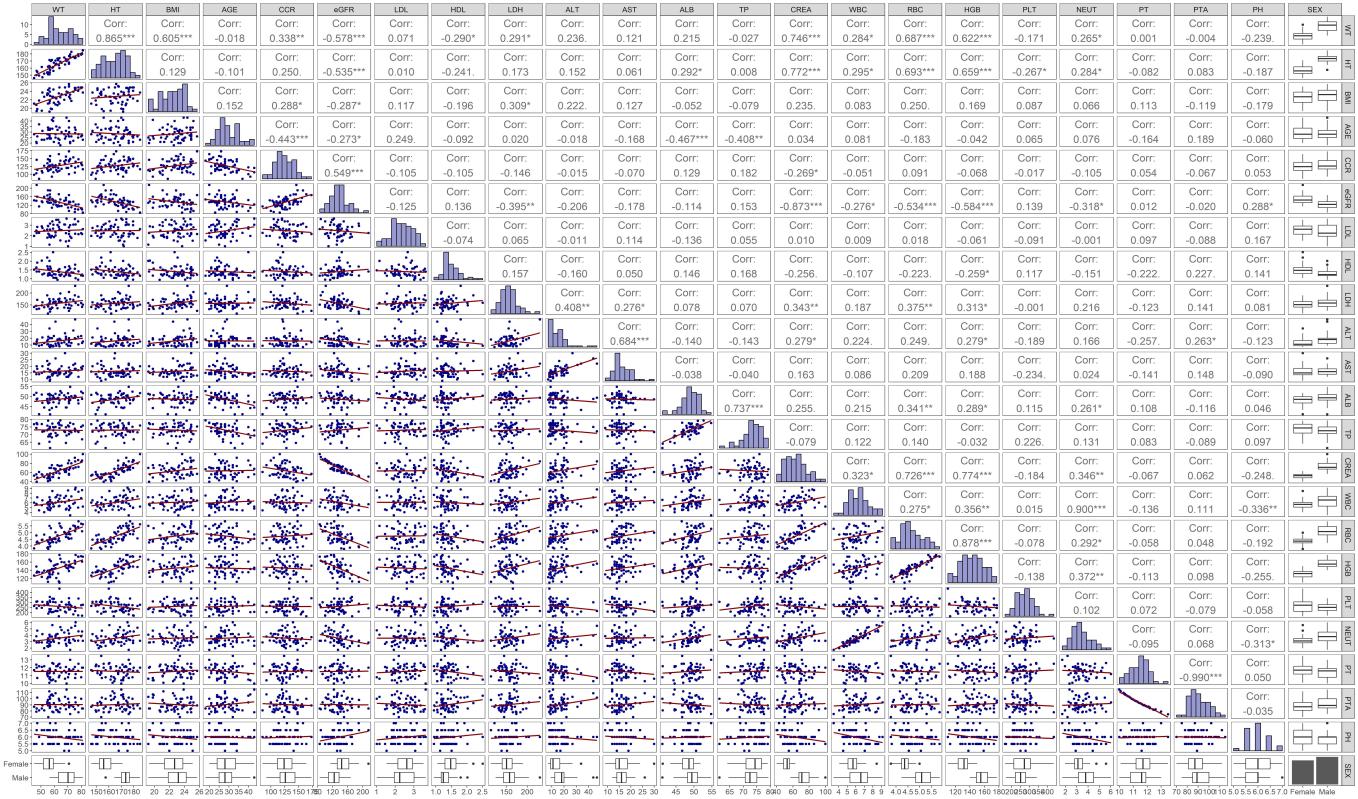


**Figure S3. Correlation between continuous and categorical covariates. A correlation coefficient greater than 0.8 is considered indicative of a correlation between covariates.**

(1) Thabit, A. K.; Crandon, J. L.; Nicolau, D. P. Pharmacodynamic and pharmacokinetic profiling of delafloxacin in a murine lung model against community-acquired respiratory tract pathogens. *International Journal of Antimicrobial Agents* **2016**, *48* (5), 535-541, Article. DOI: 10.1016/j.ijantimicag.2016.08.012.
